# Evoked Frontal and Parietal Field Potential Signatures of Target Detection and Response Inhibition in Rats Performing an Equiprobable Auditory Go/No-Go Task

**DOI:** 10.1523/ENEURO.0055-19.2019

**Published:** 2020-01-02

**Authors:** Payal Nanda, Allyn Morris, Jessica Kelemen, Jane Yang, Michael C. Wiest

**Affiliations:** Neuroscience Department, Wellesley College, Wellesley, MA 02481

**Keywords:** coherence, cortex, event related potentials, impulse control, oscillatory synchronization, sustained attention

## Abstract

To characterize the rat as a potential model of frontal-parietal auditory processing during sustained attention, target detection, and response inhibition, we recorded field potentials (FPs) at multiple sites in medial-dorsal frontal and posterior parietal cortex simultaneously while rats performed an equiprobable auditory go/no-go discrimination task. Event-related potentials (ERPs) were calculated by averaging tone-triggered FPs across hit, miss, false alarm (FA), and correct rejection (CR) trials separately for each recording session, and five peak amplitudes (termed N1, P2, N2, P3E, and P3L) were extracted from the individual-session ERPs. Comparing peak amplitudes across different trials types indicated a statistically significant amplification of the P2 peak on hit trials that accompanies detection of the target tone prior to the behavioral go response. This result appears analogous to human ERP phenomena during auditory target discrimination. Conversely, the rat P3 responses were not associated with target detection as in the human ERP literature. Likewise, we did not observe the “no-go N2” or “no-go P3” responses reported in the human literature in association with response inhibition, which might reflect differences in task context or a difference in auditory processing between rats and humans. We also present analyses of stimulus-induced spectral power and interarea coherence to characterize oscillatory synchronization which may contribute to ERPs, and discuss possible error-related processing at the N2, P3E, and P3L peaks.

## Visual Abstract

**Figure:**
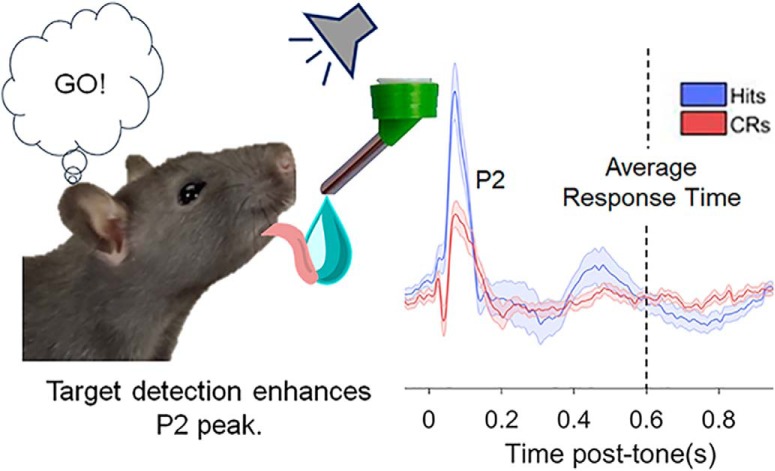

## Significance Statement

Our results constrain potential neural models of sustained attention and auditory discrimination in rat cortex. To our knowledge, our study is the first to unambiguously support that the rat P2 auditory event-related potential (ERP) component is amplified by target detection as distinct from response production. This validates that our experimental paradigm can be used to mechanistically probe the cellular basis of the ERP, and potentially could reveal how ERP phenomena are disrupted in multiple neuropsychiatric disorders. Our results complement those of “active oddball” studies in which a neural response potentially related to target detection or response activation may be confounded with automatic rare-tone response amplification.

## Introduction

In humans, attention is generally associated with the amplification of N1, P2, and P3 components of cortical event-related potentials (ERPs) as compared with passive or ignored stimulation contexts (Picton and Hillyard, 1974; Crowley and Colrain, 2004; Linden, 2005; Polich, 2007). Go/no-go tasks offer a window into the functional involvement of these ERP peaks in target detection and response production or inhibition. In go/no-go tasks, a positive P2 peak around 200 ms post-stimulus that tends to be larger on go trials has been suggested to underlie response activation (Gajewski and Falkenstein, 2013; Borchard et al., 2015), but other studies found the P2 to be larger on no-go trials and attributed it to stimulus classification processing (Crowley and Colrain, 2004). Similarly, a later positive parietal “go P3” peak (i.e., larger on go trials) has been suggested to underlie target detection (Picton and Hillyard, 1974; Linden, 2005; Polich, 2007) or response production (Pfefferbaum et al., 1985).

Rats passively exposed to auditory stimuli exhibit a vertex ERP response comparable to the human N100-P200 complex (Knight et al., 1985); we will refer to these peaks as the N1 and P2. More recent studies revealed a P3 ERP response (Yamaguchi et al., 1993; Imada et al., 2013) that shares some properties with the frontal-central P3a that signals novelty in humans oddball paradigms (Courchesne et al., 1975; Squires et al., 1975a,b; Bledowski et al., 2004; Polich, 2007). This response is distinct from a later target detection-related P3b potential with a central-parietal distribution (Linden, 2005; Polich, 2007). ERP studies of rats performing auditory “active oddball” tasks in particular (Shinba, 1997, 1999; Sambeth et al., 2003, Hattori et al., 2010) have generally supported that active engagement in the task (i.e., attention) dramatically amplifies ERP responses at P2 and P3 peaks in a manner roughly comparable to their counterparts in humans. However, target stimuli were rare (i.e., “oddballs”) compared to distractor stimuli in these studies, and it is not clear to what extent automatic rare-tone response amplification (i.e., oddball responses) contributed to their results.

To characterize ERP correlates of target detection in rats, constrain their neural mechanisms, and further characterize the rat brain as a model of executive functions, we simultaneously recorded FPs at multiple sites in medial-dorsal frontal cortex and posterior parietal cortex in rats performing an equiprobable auditory go/no-go discrimination task. Target and distractor tones were equiprobable to eliminate confounding rare-tone response amplification (i.e., oddball) effects. In particular, we sought to test whether rat cortical P2 or P3 ERP responses are modulated by target detection or motor response activation as in humans. To compare evoked response components on different trial types, we defined three positive-going ERP peaks (P2 usually between 50 and 100 ms post-tone, P3E typically near 200 ms, and P3L after 400 ms) and two negative-going ERP peaks (N1 around 30 ms post-tone, and N2 between P2 and P3L).

A secondary objective of this study was to test whether “no-go N2” or “no-go P3” responses comparable to those observed in human ERPs (Pfefferbaum et al., 1985; Smith et al., 2008; Gajewski and Falkenstein, 2013) could be observed in rats performing an equiprobable auditory go/no-go task, as potential neural correlates of cognitive or motor response inhibition (Smith et al., 2008).

## Materials and Methods

### Animals

A total of thirteen male Long–Evans rats, *Rattus norvegicus* (500–700 g, Charles River Laboratories), were used for this study. Nine rats comprised our main go/no-go dataset, while two rats were used for control recordings under passive auditory stimulation, and two rats (noted below) did not produce viable data. Rats were housed in pairs before multi-electrode array implantation surgery and individually after surgery in a dedicated animal care facility on a 12/12 h light/dark schedule (lights on at 6 A.M./off at 6 P.M.) with ad libitum access to food. Rats were allowed limited water access during the week to encourage task participation and free water access for 15–20 min post-training and over the weekend. The weights of each rat were recorded before each training session, and a rat was returned to ad libitum water if their weight fell below 85% of their lifetime maximum weight. All methods were performed in accordance with the Wellesley College animal care committee’s regulations.

### Go/no-go task

**Figure 1.**
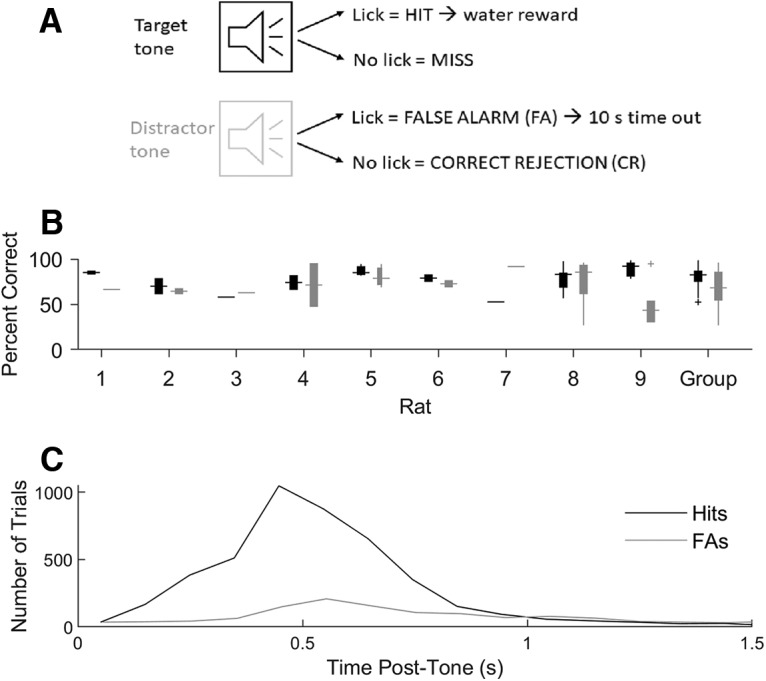
Rat behavior. ***A***, Auditory go/no-go discrimination task: rats were presented equiprobably with either a target or distractor tone, followed by a 3-s response window. Licks in response to the target tone (a hit) result in a squirt of water, while failing to lick (a miss) forfeits the opportunity for water reward. Licking in response to the distractor tone is deemed a FA and results in a 10-s penalty period. A CR refers to when the rat successfully refrains from licking after the distractor tone. Licks during the ITI were punished with a 10-s time out. On hit trials, rats were given a 3-s grace period after the response period, during which additional licks would not be penalized. ***B***, Distribution of single-session behavioral performance (percentage correct) of each rat on target (go, black box plots) and distractor (no-go, gray box plots) trials, shown by the median performance (horizontal lines), the typical range (vertical boxes), and putative outlier sessions (plus signs). The right group column plots the distribution of performances for the whole group of nine rats. ***C***, RT histogram of first-lick latencies on hit (black line) and FA (gray line) trials cumulated over 30 go/no-go sessions in nine rats.

An auditory sustained attention task was designed to capture goal-driven, top-down attention (Fig. 1). ABET-II software (Lafayette Instruments, Inc.) was used to program the task and generate pure tones. Rats were placed into standard operant chambers (80003NS, Lafayette Instruments, Inc.) and presented with either a target (3000 Hz) tone for 80 ms during go trials or a distractor (1500 Hz) tone for 80 ms during no-go trials. The sound pressure level of both tones was 60 dB (i.e., relative to the human approximate threshold of 20 μPa). Both tones were equally probable and ABET-II randomly selected the intertrial interval (ITI) to be 1, 2, or 3 s. A rat licking prematurely during the ITI period resulted in a 10-s penalty period during which the rat would not receive another trial. If the rat continued to lick during the ITI period, it would once again enter the penalty period.

Following a lick-free ITI period, an 80-ms tone played to initiate either a go or no-go trial. For both trial types, the rats had 3 s to respond appropriately. During *go* trials, the rats were trained to lick in response to a target tone (a hit trial) to receive a water reward. A new ITI period would then start 6 s after the target tone (including the 3-s response window and an additional 3-s grace period during which further licking was not penalized). If a rat failed to lick after a target tone (a miss trial), it did not receive the water reward but incurred no further penalty before entering a new ITI period. During no-go trials, the rats were trained to refrain from licking in response to distractor tones [a correct rejection (CR) trial] for 3 s before entering a new ITI period. If a rat licked within 3 s after a distractor tone [a false alarm (FA) trial], it entered into the 10-s penalty period. A training session lasted for 40–60 min. Rats typically took 40–50 training sessions to reach 80% correct on both go and no-go trials, but we observed rats reach stable performance at this criterion level in as few as 12 training sessions.

### Multi-electrode array implantation surgery

**Figure 2.**
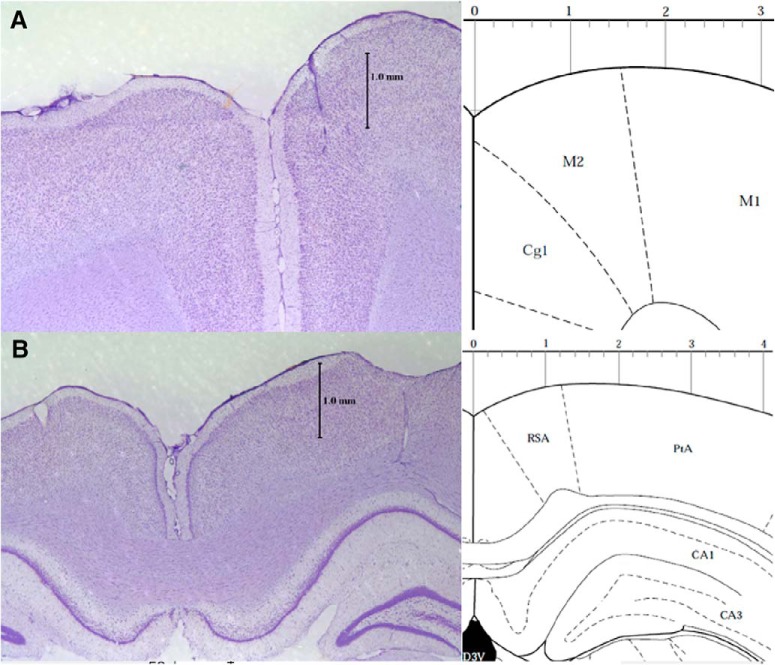
Histology. Representative Nissl-stained coronal sections (left panels) showing electrode penetrations in the right hemisphere (next to the 1-mm vertical calibration bar) of dorsomedial frontal (***A***) and posterior parietal (***B***) cortex. The right panels show diagrams from the Paxinos and Watson rat brain atlas (Paxinos and Watson, 1997) with their designations for the cortical areas 2.7 mm anterior to bregma (in ***A***; Cg1 is cingulate cortex, M2 and M1 are secondary and primary motor cortex) and 4.16 mm posterior to bregma (in ***B***; PtA is parietal association cortex, RSA is retrosplenial agranular cortex, and CA1 and CA3 are fields of the hippocampus).

A total of 10 of 11 rats successfully learned the go/no-go task (80% correct for both go and no-go trials) and underwent surgery for electrode implantation. Rats were removed from the water-deprivation schedule at least 2 d before surgery and given bacon-flavored painkiller tablets daily to familiarize them with the flavor of the tablets. Rats were placed in a stereotaxic apparatus with atraumatic ear-bars during surgery and sedated and anesthetized using isoflurane (1–2% in O_2_) and locally anesthetized by bupivacaine (0.125%, 2 mg/kg, 0.16 ml/100 g) before incision. 32-microelectrode arrays (Innovative Neurophysiology, Inc.) were implanted in the right frontal (2.0 mm anterior to bregma, 0.75 mm right from midline, and 1.5 mm beneath the brain surface) and right parietal (4.15 mm posterior to bregma, 3.5 mm right from midline, and 1.2 mm beneath the brain surface) cortices and secured with dental cement. A 2 × 16 array was placed in the frontal cortex, and a 4 × 8 array was placed in the parietal cortex. Both arrays had a row spacing of 300 μm and an interelectrode spacing of 150 μm apart (resulting in anterior-posterior spans of ∼2.25 and 1.05 mm for the frontal and parietal arrays, respectively). Our electrodes were 35 μm in diameter with “micro polished” tips (Innovative Neurophysiology, Inc.) resulting in ∼962 μm^2^ of exposed tip area and impedance between 500 and 750 kΩ at 1 kHz. The arrays were grounded by wires attached to skull screws in the left frontal, left parietal, and right occipital lobes. Following surgery, rats were given bupivacaine (0.125%, 2 mg/kg, 0.16 ml/100 g) for pain relief up to 48 h post-surgery. Rats were weighed and monitored for pain daily for one week after surgery and were allowed free access to water and food during recovery. Five of the nine brains (after discarding an outlier animal described below) included in the FP dataset described below were processed histologically to confirm electrode penetration in the target areas. Forty-micrometer coronal sections from frontal and parietal cortex were Nissl-stained and electrode penetration tracks (Fig. 2) were observed in both frontal and parietal cortices in each of the five brains.

### Electrophysiological recording

Electrophysiological recordings were performed after at least one week of recovery from implantation surgery. Rats were briefly anesthetized using isoflurane (4% in O_2_) to insert the recording head-stage. Recordings did not begin until the rats exhibited normal motor control. FP activity referenced to ground was recorded from the frontal and parietal cortices during the go/no-go task using a Cerebus Data Acquisition System (Blackrock Microsystems) with a 1000-Hz sampling rate. A 150-Hz low-pass filter was applied to remove high-frequency artifacts and noise. For analysis, the FP data were transferred to MATLAB using NeuroExplorer.

### Data pre-processing

All data analysis was conducted in MATLAB on PC computers running Windows 10, and all sessions underwent pre-processing before ERP analysis as described below. The analysis code described in this paper is freely available online at https://repository.wellesley.edu/neurosciencefaculty/. We only included a session in our dataset if a χ^2^ analysis supported that the proportion of go responses on target trials was significantly different from the proportion of go responses on distractor trials (*p* < 0.001). Due to RAM limitations of our analysis computers, we used 32 FP channels distributed across both arrays (avoiding neighbor electrodes) rather than the full set of 64. FP trials were defined in a 1.5-s window starting 0.5 s before the tone and ending 1 s after tone onset. Each session underwent artifact rejection analysis to discard trials with flat lines or extreme FP signals exceeding 600 μV. If >10% of trials were found to exceed this cut value on a particular electrode, then that electrode was omitted from the analysis. In addition, FPs were bandpass filtered between 0.5 and 50 Hz. After discarding four sessions from one outlier animal described below and five other sessions contaminated by artifact, the cleaned dataset comprised a total of 10,578 trials recorded across 30 sessions in nine animals, with an average of 353 trials per session being recorded from an average of 13 frontal channels and 15 parietal channels. All but three of the 30 sessions had more than five surviving channels on each of its arrays after artifact rejection.

### ERP analysis

FP signals were averaged across surviving FP channels on each array separately to produce frontal and parietal average FPs, which were then averaged across trials within each trial type (hit, miss, CR, FA) to produce frontal and parietal ERPs for each session. Grand average ERP plots for each trial type were produced by averaging ERPs across 30 sessions from nine rats. Each session in the grand average was weighted according to the number of trials in the session. Our trained animals generally produced greater numbers of correct than incorrect trials, with average numbers of hit, CR, miss, and FA trials per session being 153, 117, 32, and 50, respectively. Five (4) of 30 sessions had fewer than 10 remaining miss (FA) trials after rejecting suspected artifactual trials as described above.

ERP component analysis was also performed for individual sessions to determine peak amplitudes and latencies for each trial type. The N1, P2, N2, early P3 (P3E), and late P3 (P3L) peaks were defined as the greatest positive-going or negative-going peak found within time windows determined by visual inspection of the grand average ERP plots and individual session ERPs (see Results). The P2 peak was identified first as the highest peak (local maximum) between 50- and 150-ms post-tone onset. The P3E was identified next as the highest peak between the P2 latency and 365-ms post-tone. Our main results (see Results) were unchanged when we re-ran our analysis using an alternate P3E definition that enforced a 60-ms gap between the P2 and P3E (data not shown). The P3L was defined as the highest peak between 350 and 1000 ms post-tone (and in every case was a distinct peak from the P3E). The negative-going N1 peak was identified as the lowest trough (local minimum) after 10-ms post-tone onset but before the P2 latency. If we detected no trough in that window, we extended the N1 search to smaller positive latencies. Finally, the N2 was defined as the lowest trough between the P2 and late P3L latencies, which could appear before or after the P3E peak. The peak latencies of one animal were consistently delayed in comparison to the rest of the dataset (e.g., P2 around 200 ms rather than before 100 ms) so this outlier animal was omitted from the analysis.

### Statistical analysis of ERP component amplitudes and latencies

A three-factor ANOVA was used to examine the effect of brain region (frontal or parietal), trial type (hit, miss, FA, or CR), and peak (N1, P2, N2, P3E, or P3L) on ERP component amplitudes and latencies. All three factors were within-subject repeated measures. Tukey’s HSD test was used for *post hoc* tests. Effects with *p* < 0.05 were considered statistically significant.

### Spectral analysis of single-trial FPs

To characterize induced oscillatory activity that might relate to the ERP components we measured, we calculated spectrograms with a sliding 200-ms window in 50-ms steps, comprising 0.2 s before to 1 s after the tone, using the multitaper Fourier transform approach implemented by the Chronux Spectral Analysis Toolbox (Bokil et al., 2007). The time-bandwidth product and number of tapers were set to [5 9] for spectrogram calculations as well as coheregram calculations described below. With a 200-ms analysis window and a 1000-Hz sampling rate, these parameters result in spectra with frequency bins ∼1-Hz wide. We focused our analysis on frequencies up to 20 Hz. We calculated spectrograms for each FP channel on each trial, and then averaged over frontal and parietal electrodes separately to produce frontal and parietal average spectrograms for each trial. These were then averaged over trials to produce average induced (i.e., non-phase locked to the tone) frontal and parietal spectrograms for each trial type in each session. These single-session-spectrograms were then averaged across sessions to produce grand average frontal and parietal spectrograms for each trial type.

We also calculated frontal-parietal coheregrams to characterize induced rhythmic coordination of activity across frontal and parietal cortices, again using a 200-ms window sliding in 50-ms steps. Interarea coheregrams were calculated for every frontal-parietal electrode pair on every trial, averaged across interarea electrode pairs, and then averaged over trials of each trial type to produce single-session coheregrams. We averaged the single-session coheregrams to generate grand average induced interarea coheregrams for each trial type.

To distinguish regions in the time-frequency plots (spectrograms and coheregrams) in which higher spectral power or coherence was significantly different on different trial types, we used two-tailed *t* tests based on the variability across sessions.

## Results

To characterize sensory processing in the frontal-parietal cortical network of rats, we recorded FPs from micro-electrode arrays implanted in the medio-dorsal frontal and posterior parietal cortex of nine rats while they performed an equiprobable auditory go/no-go task (Fig. 1*A*). We histologically confirmed electrode penetrations into both target areas in five of the nine rats (Fig. 2).

### Behavior

Behavioral performance varied across rats and individual recording sessions as shown in Figure 1*B*, averaging 80 ± 13% (SD) correct during target trials and 68 ± 21% (SD) correct on distractor trials. In every recorded session, rats responded by licking significantly more during target trials than during distractor trials as assessed by a χ^2^ proportion comparison (*p* < 0.001), demonstrating that they were effectively discriminating target and distractor tones despite variations in performance.

On hit trials, it took rats an average of 0.6 ± 0.3 s (SD) from tone onset to produce their lick response, as compared to an average reaction time (RT) of 1.0 ± 0.7 s (SD) during FA trials. The distributions of RTs on hit and FA trials were similar (Fig. 1*C*).

### ERPs

**Figure 3.**
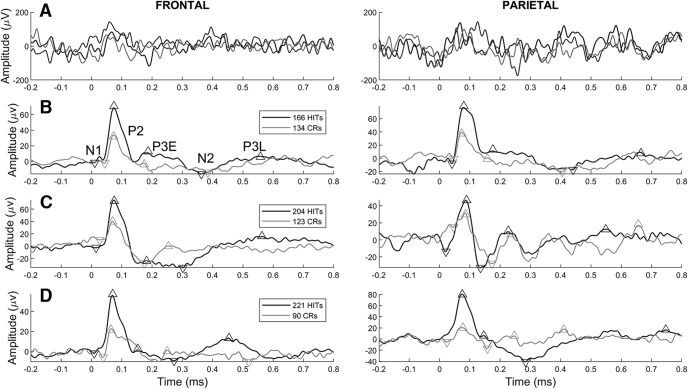
Single-trial FPs and single-session frontal and parietal auditory ERPs. ***A***, Single-electrode field potentials (FPs) recorded in frontal (left panel) and parietal (right panel) cortex on three example hit trials. ***B****–****D***, Single-session hit (black traces) and CR (gray traces) ERPs (in microvolts) recorded in frontal (left panels) and parietal (right panels) cortex of a different rat in each row. The ERPs shown are averaged frontal and parietal FPs referenced to the onset of the target or distractor tone at *t* = 0 on the horizontal axis. Upwards and downwards triangles label the approximate latencies and amplitudes of the N1, P2, N2, P3E, and P3L peaks identified by our automated analysis.

Figure 3*A* shows example single-trial FPs recorded from a frontal and parietal array. To characterize stimulus-locked frontal-parietal auditory processing, FP responses aligned to the onset of target (go) or distractor (no-go) tones were averaged across frontal and parietal recording electrodes and across hit, miss, CR, and FA trials separately to generate frontal and parietal ERPs for each trial type from each recording session. In particular, we aimed to test potential roles of the P2 and P3 (E or L) peaks in target detection, and possible roles of the N2 or P3 (E or L) peaks in response inhibition.

On hit trials a target was correctly detected, as indicated by a licking response, while a CR was characterized by withholding of a licking response following a distractor tone. Figure 3*B–D* compare hit and CR ERPs from frontal and parietal cortex during three example recording sessions in three different rats. These examples illustrate the variability of ERPs across rats, but also show a consistent pattern that motivates our definitions of five ERP peaks for analysis: two negative-going peaks called N1 and N2, and three positive-going peaks that we label as P2, P3E (early P3) and P3L (late P3) as shown in Figure 3*B* (see Materials and Methods).

Note that the N2 can appear before or after the P3E, depending on which side of the sustained negativity is deeper. Figure 3*B* shows a case where the N2 appears after the P3E (clearest in the frontal hit ERP in the left panel), whereas Figure 3*C* shows an example in which the CR ERP (in the right panel) has a deeper negativity (the N2) before the P3E. The example in Figure 3*D* shows that in some ERPs the P3E is not visible at all or can appear as a “hump” on the downslope of the P2 (in the right panel).

### Hit versus CR grand ERPs

**Figure 4.**
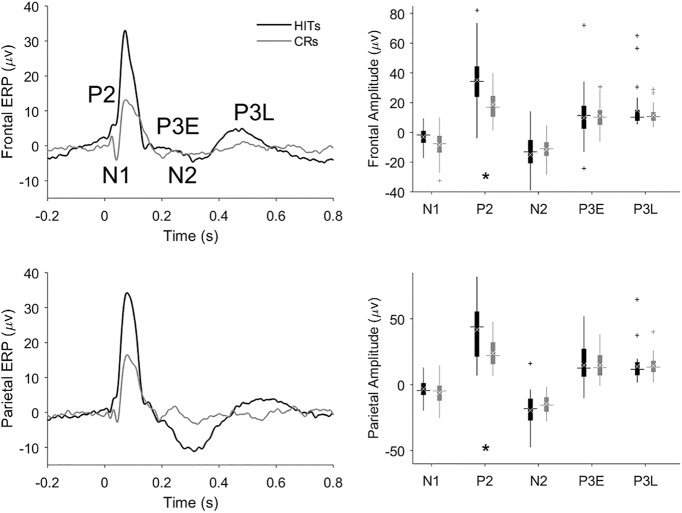
Comparison of hit and CR trial grand average ERPs and component amplitudes. Grand average ERPs and peak amplitudes from frontal and parietal cortex are shown in the upper and lower panels, respectively. FPs from the frontal or parietal cortex were averaged to produce ERPs (left panels) for hit (black traces and black box plots) and CR trials (gray traces and gray box plots). The ERPs were referenced to the onset of the tone. We used the peak amplitudes of the session ERPs to compute the component amplitudes for each session whose distribution is shown by the box plots in the right panels. Horizontal lines show median peak amplitude, white xs denote the mean amplitude, and plus signs denote putative outliers outside 1.5× the interquartile range. In calculating the grand average ERPs, the single-session ERPs were weighted according to each session’s number of trials to give greater weight to the more statistically reliable (i.e., higher-n) ERPs. The asterisk indicates a significant target detection-related amplification of the P2 peak on hits compared to CRs (Tukey’s HSD, *p* = 0.006)^f^.

To capture stimulus-locked processing components that were consistent across animals, we averaged single-session ERPs to produce grand average ERPs for each trial type. Grand average hit and CR ERPs are compared in the left panels of Figure 4. The peaks identified in individual sessions (Fig. 3) are visible in the frontal and parietal grand hit ERPs (Fig. 4, left panels). The most prominent effect suggested by comparing the hit and CR ERPs is the dramatic amplification of the P2 on hit trials in both frontal and parietal cortex as compared to CR trials. The frontal and parietal N2 and P3L peaks also tend to be larger on hit trials versus CRs. The N1 is the only component that trends toward being larger on CRs as compared to hits in the grand ERPs. The right panels of Figure 4 depict the range of peak amplitudes extracted from individual-session ERPs.

### Statistical analysis of peak amplitudes

To better interpret the trends observed in the hit-CR grand ERP comparison, we performed a three-way repeated-measures ANOVA of individual-session ERP peak amplitudes, incorporating factors: brain region × trial type × peak. We found a main effect of peak on amplitude but did not pursue this further as our primary objective was to determine the effect of trial type on each ERP component. The brain region × peak interaction was significant (*F*_(4,116)_ = 3.1, *p* = 0.02; Table 1)^a^. *Post hoc* Tukey tests found the parietal N2, P3E, and P3L peaks to be significantly larger in amplitude than their frontal counterparts (*p* = 0.03, *p* = 0.04, *p* = 0.01)^b,c,d^.

**Table 1.** Statistical results

	Data structure	Type of test	*p* value	Power
a	Normal	ANOVA brain region × peak interaction	0.02	0.80
b	Normal	Tukey’s HSD	0.03	0.37
c	Normal	Tukey’s HSD	0.04	0.33
d	Normal	Tukey’s HSD	0.01	0.46
e	Normal	ANOVA trial type × peak interaction	2 × 10^–5^	0.99
f	Normal	Tukey’s HSD	0.006	0.71
g	Normal	Tukey’s HSD	0.0009	0.85
h	Normal	Tukey’s HSD	0.3	0.22
i	Normal	Paired *t* test	0.04	0.81
j	Normal	Tukey’s HSD	0.003	0.76
k	Normal	Tukey’s HSD	0.03	0.52
l	Normal	Tukey’s HSD	4 × 10^–6^	0.99
m	Normal	Tukey’s HSD	0.04	0.49
n	Normal	Levene’s test for equal variances	0.009	0.82
o	Normal	Levene’s test for equal variances	0.004	0.83

The letters in the left column are superscripts labeling each statistical result reported in the text. The second and third columns from left describe the data distribution and statistical test used in each case. The two right-most columns list *p* values reported in the text and the estimated *post hoc* power corresponding to each *p* value. We estimated *post hoc* power of *F* tests using Equation 4 from Lenth (2007) cited in Pek and Park (2019) and estimated *post hoc* power of the pairwise *post hoc* tests as the *post hoc* power of the equivalent *t* test using the MATLAB function *sampsizepwr.m*.

Our ANOVA also revealed a significant trial type × peak interaction (*F*_(12,348)_ = 3.8, *p* = 2 × 10^−5^)^e^ that justifies the *post hoc* test results reported below for each peak.

### N1 peak amplitudes

Pairwise *post hoc* tests comparing N1 amplitude on different trial types did not reveal any significant differences.

### P2 peak amplitudes

**Figure 5.**
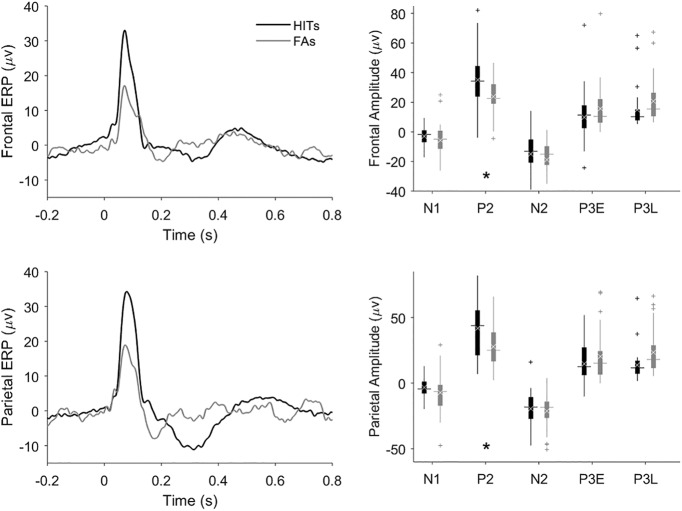
Hit versus FA ERPs and component amplitudes. As in Figure 4, Grand average ERPs and peak amplitudes from frontal and parietal cortex are shown in the upper and lower panels, respectively. FPs from the frontal or parietal cortex were averaged to produce ERPs (left panels) for hit (black traces and black box plots) and FA trials (gray traces and gray box plots). The ERPs were referenced to the onset of the tone. We used the peak amplitudes of the session ERPs to compute the component amplitudes for each session whose distribution is shown by the box plots in the right panels. Horizontal lines show median peak amplitude, white xs denote the mean amplitude, and plus signs denote putative outliers outside 1.5× the interquartile range. In calculating the grand average ERPs, the single-session ERPs were weighted according to each session’s number of trials to give greater weight to the more statistically reliable (i.e., higher-n) ERPs. The asterisk indicates a significant amplification of the P2 peak on hits compared to FAs (Tukey’s HSD, *p* = 0.0009)^g^.

**Figure 6.**
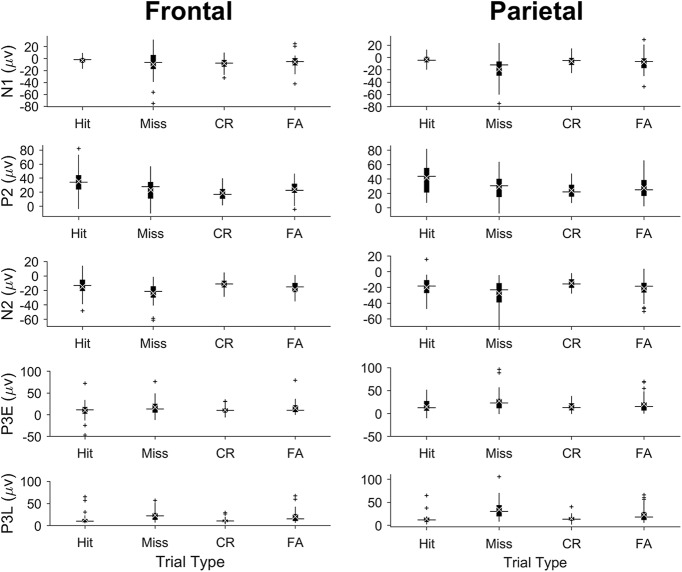
Single-session ERP-peak amplitudes by trial type. Each row presents box plots of peak amplitudes (in microvolts) extracted from single-session ERPs for each trial type (hits, misses, CRs, and FAs). Peak amplitudes from frontal and parietal ERPs are shown in the left and right panels, respectively. As in previous box plots horizontal lines show median peak amplitude, white xs denote the mean amplitude, and plus signs denote putative outliers outside 1.5× the interquartile range.

**Figure 7.**
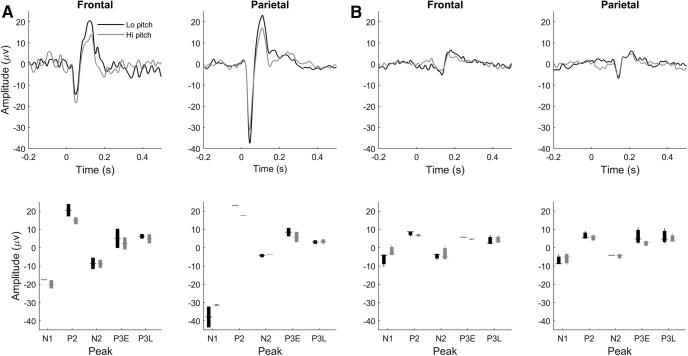
ERP responses to equiprobable passive presentation of lower-pitched and higher-pitched tones equivalent to the distractor and target tones, respectively, in the active go/no-go task. ***A***, ***B***, ERPs and peak amplitudes for two different rats. Upper panels depict frontal and parietal ERPs recorded in response to the lower pitch (black traces) or higher pitch (gray traces), while lower panels show box plots (with conventions as in previous figures) of peak amplitudes for lower pitch (black) or higher pitch (gray) ERPs. The lower pitch (distractor in the active context) tends to elicit a larger P2 peak than the higher pitch; this difference was significant in the pooled data (paired *t* test, df = 9, *p* = 0.04)^i^. In the passive context, rats were not water restricted and had no access to a lick tube.

A *post hoc* Tukey test found the amplification of the P2 peak on hits compared to CRs (noted above from the grand ERPs in Fig. 4) to be highly significant (*p* = 0.006)^f^. Amplification of the P2 peak on hits as compared to CRs might reflect target identification or behavioral (motor) response activation, which both occur on hits but not CR trials. To constrain this interpretation, we compared hit to FA ERPs (Fig. 5) because both hits and FAs involve a motor response (lick), but actual target detection occurs only on hit trials. The frontal and parietal P2 peaks are significantly larger on hits than FAs in both the grand ERPs and component analysis (Tukey’s HSD, *p* = 0.0009)^g^, suggesting the P2 enhancement on hit trials cannot be solely attributed to response activation or neural correlates of licking for water. The P2 tended to also be larger on hits than miss trials (Fig. 6), though this difference was not significant (*p* = 0.3)^h^. It is possible that the P2-evoked response to the higher frequency target is larger than the response to the lower frequency distractor independent of task context, though previous passive recordings did not reveal any frequency tuning of the P2 component (Knight et al., 1985). To further constrain this possibility, we recorded ERP responses in two rats (distinct from all rats in the active go/no-go dataset) to equiprobable passive presentation of the two tones used in our active task (Fig. 7). In both rats the P2 amplitude in response to the lower pitch (1500 Hz, the distractor in the active go/no-go task) tended to be larger than the response to the higher pitch (3000 Hz, the target in the active task). This difference was significant in the pooled data (paired *t* test, degrees of freedom (df) = 9, *p* = 0.04)^i^.

### N2 peak amplitudes

**Figure 8.**
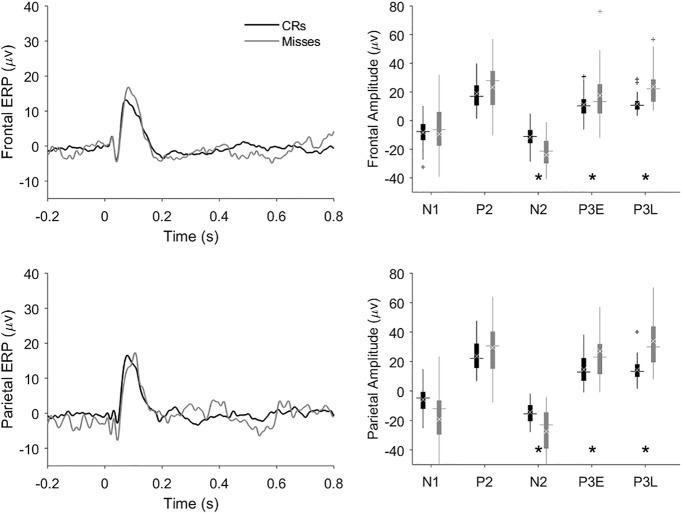
Comparison of CR and miss trial ERPs and component amplitudes. As in Figure 4, Grand average ERPs and peak amplitudes from frontal and parietal cortex are shown in the upper and lower panels, respectively. FPs from the frontal or parietal cortex were averaged to produce ERPs (left panels) for CR (black traces and black box plots) and miss trials (gray traces and gray box plots). The ERPs were referenced to the onset of the tone. We used the peak amplitudes of the session ERPs to compute the component amplitudes for each session whose distribution is shown by the box plots in the right panels. Horizontal lines show median peak amplitude, white xs denote the mean amplitude, and plus signs indicate putative outliers outside 1.5× the interquartile range. In calculating the grand average ERPs, the single-session ERPs were weighted according to each session’s number of trials to give greater weight to the more statistically reliable (i.e., higher-n) ERPs. Asterisks indicate significantly greater peak amplitude on miss trials compared to CRs at the N2 (Tukey’s HSD, *p* = 0.003)^j^, P3E (*p* = 4 × 10^−6^)^l^, and P3L (*p* = 0.04)^m^ peaks.

The grand hit ERPs show a negative dip (the N2; especially in parietal cortex) that is essentially absent from the grand CR ERPs (Fig. 4), providing no support for a no-go N2 in rats, at least in the context of the equiprobable go/no-go task. To further investigate potential correlates of response inhibition we compared CR ERPs to miss trial ERPs (Fig. 8). Since no licking response is executed in either CR trials or miss trials, any difference between miss and CR ERPs could reflect active response inhibition. Though the difference is not visible in the grand ERPs of Figure 8, the N2 peak extracted from individual sessions was significantly larger (i.e., more negative) on miss trials as compared to CR trials (Tukey’s HSD, *p* = 0.003)^j^.

### P3E peak amplitudes

The P3E amplitude on miss trials was significantly larger than on CR trials (Fig. 8; Tukey’s HSD, *p* = 0.03)^k^.

### P3L peak amplitudes

The P3L amplitude was significantly larger on miss trials than CR trials (Fig. 8; *p* = 4 × 10^−6^)^l^ and FA trials (Fig. 8; *p* = 0.04)^m^.

To further constrain interpretation of the amplification of miss trials noted on the N2, P3E, and P3L peaks, we performed a statistical comparison of ERP variances on different trial types. Levene’s test of equal variances revealed a significant difference among the FP amplitude variances of the four trial types (quadratic Levene’s test, *F*_(3,236)_ = 3.9, *p* = 0.009)^n^. A follow-up test showed that variances on miss and FA trials were significantly larger than on hit and CR trials (quadratic Levene’s test, *F*_(1,238)_ = 8.5, *p* = 0.004)^o^.

### Peak latency analysis

**Figure 9.**
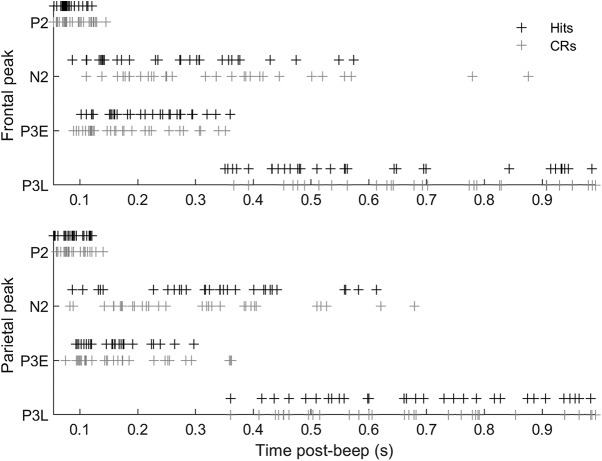
Distribution of ERP peak latencies on hit (black crosses) and CR (gray crosses) trials. Each cross marks the latency of the respective peak (labeled on the vertical axis) in the ERP from one of 30 recording sessions (from nine rats) in our dataset. The N1 peak was not identified in every session and is omitted from the figure.

Figure 9 shows the range of latencies of each peak across sessions. A three-way ANOVA performed on the latencies of the ERP component peaks did not reveal any significant effect of brain region or trial type on peak latencies.

### Spectral analysis: spectrograms

**Figure 10.**
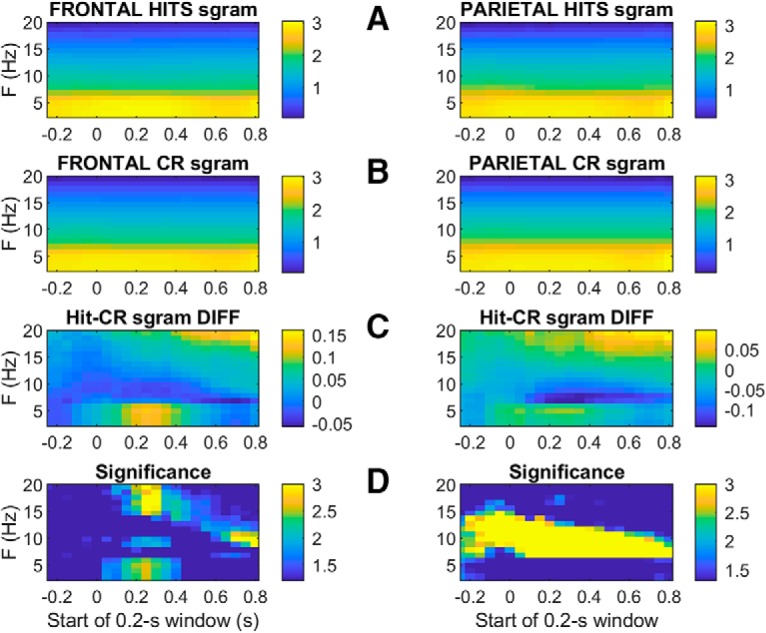
Average Hit and CR spectrograms from frontal (left panels) and parietal (right panels) electrodes, aligned to tone onset at *t* = 0. Hit and CR spectrograms are shown in rows ***A***, ***B***, respectively. Row ***C*** shows the difference between average H and CR spectrograms (hit minus CR), such that the light blue and yellow time-frequency bins indicate greater hit power than CR power, and dark blue bins indicate greater CR power than hit power. The color axes in rows ***A–C*** show power transformed to arbitrary logarithmic units to allow smaller-amplitude features at higher frequencies to be visible. Row ***D*** shows the significance of a two-tailed *t* test comparing spectral power on hits and CR trials at each time-frequency bin, based on the variability of the trial-averaged power across sessions (*n* = 30). The statistical significance of the hit-CR difference at each time-frequency bin is plotted as log10(1/p) such that differences from dark blue on the color scale indicates significant differences above the 95% confidence level, while light blue (color scale = 2) and yellow (color = 3) indicate significance with 99% and 99.9% confidence, respectively [i.e., since log10(1/0.001) = 3; not corrected for multiple comparisons; the *t* tests were based on raw trial-averaged spectral powers in units of square microvolts per Hz, not the logarithmically transformed values displayed in rows ***A–C***].

To investigate whether the ERP peaks we observed are related to the onset of events within particular frequency bands, we calculated spectrograms quantifying oscillatory power for every electrode in a sliding 200-ms window relative to tone onset on every trial. We averaged these separately across frontal and parietal electrodes and across each trial type separately to generate frontal and parietal average spectrograms for each trial type for each session. The single-session-spectrograms were averaged to produce grand average frontal and parietal spectrograms. Figure 10 compares hit to CR spectrograms. In both frontal and parietal cortices both trial types exhibit typical power spectra with relatively large power at lower frequencies, and both show a modest increase in low-frequency power induced by the tone at *t* = 0 (Fig. 10*A*,*B*). The difference spectrograms in Figure 10*C* (hit spectrogram – CR spectrogram) reveal distinct phenomena in different frequency bands. The tone-induced power between 7 and 14 Hz tends to be greater on CRs than hits, whereas at frequencies below 7 Hz and above 14 Hz, hit trials have more power than CRs. Figure 10*D* plots the significance of these differences in terms of the p-values produced by conducting a *t* test at each time-frequency bin.

**Figure 11.**
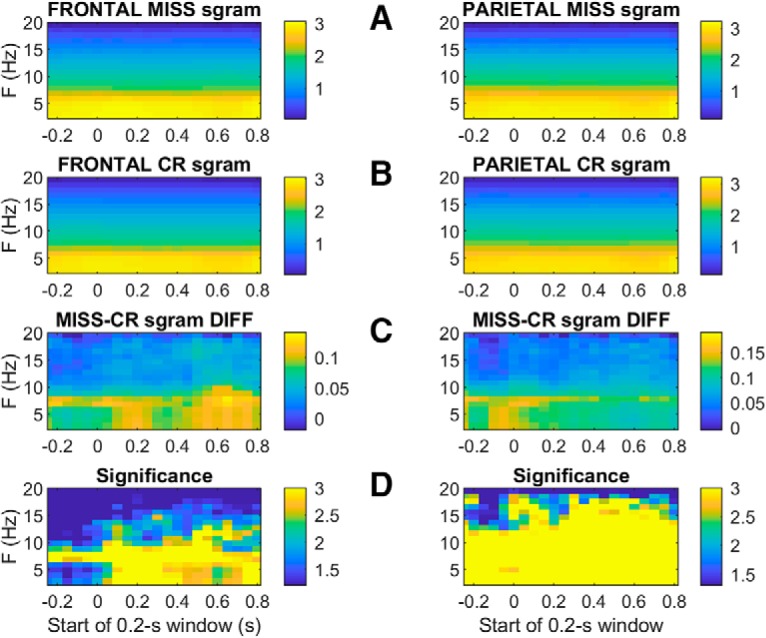
Average Miss and CR spectrograms from frontal (left panels) and parietal (right panels) electrodes, aligned to tone onset at *t* = 0. Miss and CR spectrograms are shown in rows ***A***, ***B***, respectively. Row ***C***, shows the difference between average miss and CR spectrograms (miss minus CR), such that the light blue and yellow time-frequency bins indicate greater miss power than CR power, and dark blue bins indicate greater CR power than miss power. The color axes in rows ***A–C*** show power transformed to arbitrary logarithmic units to allow smaller-amplitude features at higher frequencies to be visible. Row ***D*** shows the significance of a two-tailed *t* test comparing spectral power on hits and CR trials at each time-frequency bin, based on the variability of the trial-averaged power across sessions (*n* = 30). The statistical significance of the miss CR difference at each time-frequency bin is plotted as log10(1/p) such that differences from dark blue on the color scale indicates significant differences above the 95% confidence level, while light blue (color-scale = 2) and yellow (color = 3) indicate significance with 99% and 99.9% confidence, respectively [i.e., since log10(1/0.001) = 3; not corrected for multiple comparisons; the *t* tests were based on raw trial-averaged spectral powers in units of square microvolts per Hz, not the logarithmically transformed values displayed in rows ***A–C***].

**Figure 12.**
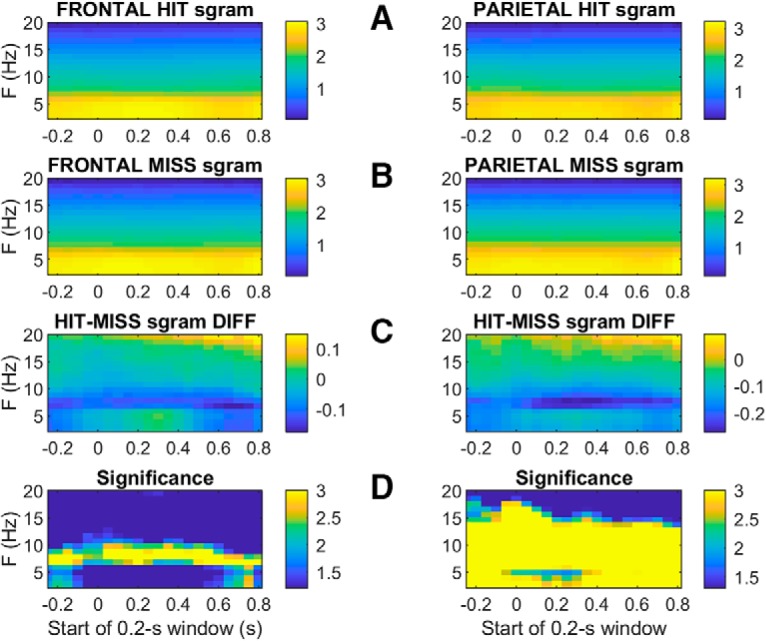
Average hit and miss spectrograms from frontal (left panels) and parietal (right panels) electrodes, aligned to tone onset at *t* = 0. Hit and miss spectrograms are shown in rows ***A***, ***B***, respectively. Row ***C*** shows the difference between average hit and miss spectrograms (hit minus miss), such that the light blue and yellow time-frequency bins indicate greater hit power than miss power, and dark blue bins indicate greater miss power than hit power. The color axes in rows ***A–C*** show power transformed to arbitrary logarithmic units to allow smaller-amplitude features at higher frequencies to be visible. Row ***D***, shows the significance of a two-tailed *t* test comparing spectral power on hit and miss trials at each time-frequency bin, based on the variability of the trial-averaged power across sessions (*n* = 30). The statistical significance of the hit-miss difference at each time-frequency bin is plotted as log10(1/p) such that differences from dark blue on the color scale indicates significant differences above the 95% confidence level, while light blue (color scale = 2) and yellow (color = 3) indicate significance with 99% and 99.9% confidence, respectively [i.e., since log10(1/0.001) = 3; not corrected for multiple comparisons; the *t* tests were based on raw trial-averaged spectral powers in units of square microvolts per Hz, not the logarithmically transformed values displayed in rows ***A–C***].

To investigate potential rhythmic contributions to the larger peak amplitudes observed on miss trials (Fig. 8), we compared miss to CR spectrograms (Fig. 11). In both frontal and parietal cortex miss trials tend to show greater power than CRs, particularly at the lower frequencies. Interestingly, the difference spectra (Fig. 11*C*) in both frontal and parietal cortices reveal a band in the θ range (near 7 Hz) with particularly high power on misses as compared to CRs, both before and after presentation of the unpredictably-timed tone. Comparing spectrograms on hit and miss trials revealed a similar excess of power near 7 Hz on miss trials as compared to hits (Fig. 12), again both before and after the tone at *t* = 0. In parietal cortex this excess power on miss trials extended to a broader range of frequencies.

### Coheregrams

**Figure 13.**
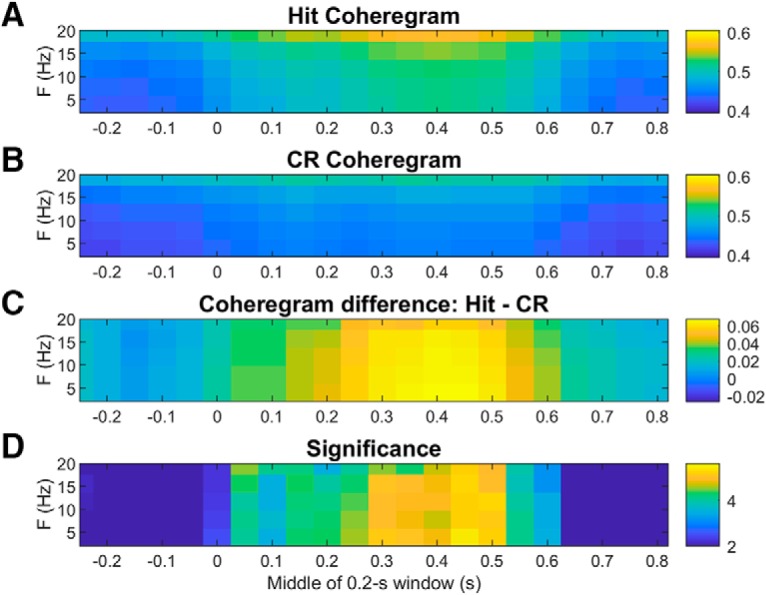
Average Hit and CR coheregrams showing frontal-parietal coherence spectra as a function of time relative to tone onset at *t* = 0. Coheregrams were averaged across trials and interarea electrode pairs, then across sessions. ***A***, Average hit frontal-parietal coheregram. ***B***, Average CR frontal-parietal coheregram. The color axes in ***A***, ***B*** depict dimensionless coherence values that can in principle range between zero and one. ***C***, Difference between hit and CR coheregrams (hit minus CR). ***D***, Time-frequency bins where the hit-CR coherence difference is significant at the 99% CL or above are plotted as log10(1/p) where p is the *p* value resulting from a two-tailed *t* test based on the variability across sessions.

**Figure 14.**
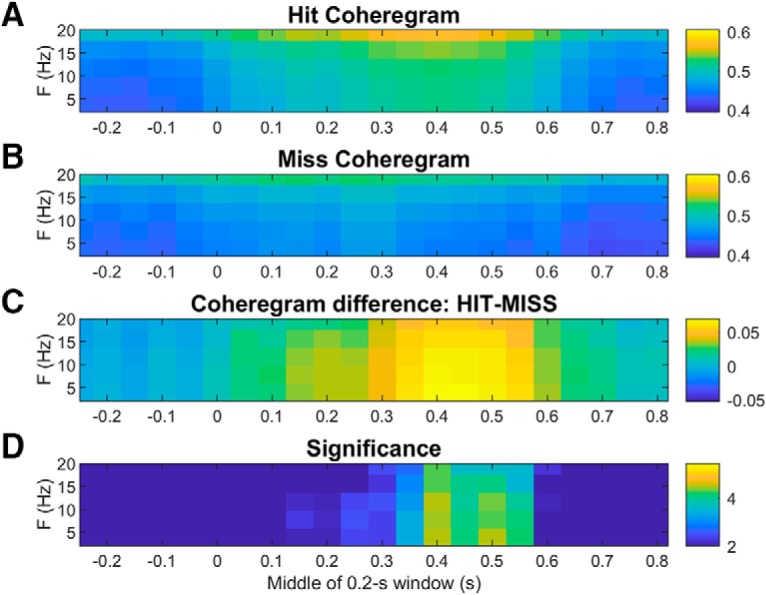
Average hit and miss coheregrams showing frontal-parietal coherence spectra as a function of time relative to tone onset at *t* = 0. Coheregrams were averaged across trials and interarea electrode pairs, then across sessions. ***A***, Average hit frontal-parietal coheregram. ***B***, Average miss frontal-parietal coheregram. The color axes in ***A***, ***B***, depict dimensionless coherence values that can in principle range between zero and one. ***C***, Difference between hit and miss coheregrams (hit minus miss). ***D***, Time-frequency bins where the hit-CR coherence difference is significant at the 99% CL or above are plotted as log10(1/p) where *p* is the *p* value resulting from a two-tailed *t* test based on the variability across sessions.

To reveal any interarea rhythmic synchronization events that might contribute to ERP components, we also calculated coheregrams for each frontal-parietal electrode pair on every trial. We averaged these over interarea electrode pairs and across trials of each type separately to generate single-session coheregrams for each trial type. We then averaged the single-session coheregrams to produce grand average coheregrams. Average coheregrams on hit and CR trials are compared in Figure 13. Unlike the power spectra displayed in the spectrograms of Figures 10-Figures 12, the interarea coherence spectra are not skewed toward the lower frequencies (Fig. 13*A*,*B*). The difference coheregram in Figure 13*C* shows greater induced fontal-parietal coherence on hit than CR trials at all frequencies up to 20 Hz. Figure 13*D* plots the significance of these differences in terms of p-values of *t* tests at each time-frequency bin. Comparing hit coheregrams to miss coheregrams (Fig. 14) shows greater induced coherence on hit trials at all frequencies up to 20 Hz, from ∼250 to 650 ms post-tone, but no significant effects before the tone. This is in contrast to the local power in each area, which was greater on misses than hits, even before the tone (Fig. 12). We also compared miss to CR coheregrams but found no significant differences (data not shown) and no sign of interarea coherence specific to the θ-frequency rhythm present on misses in each cortical area individually (Figs. 11, 12).

## Discussion

To characterize cortical ERP responses in rats during auditory target detection and behavioral response inhibition we recorded FPs from multiple sites in medial-dorsal frontal and posterior parietal cortex while rats performed an auditory go/no-go discrimination task. Lower average performance on distractor (no-go) trials as compared to target (go) trials supports that active response inhibition was required to control the impulse to lick on distractor trials.

### The P2 amplitude reflects target detection, not just response activation

We observed an amplification of the P2 amplitude on hit trials as compared to CRs, consistent with the involvement of P2 activity in target detection or initiation of a licking response, which both occur during hit but not CR trials. That we also observed an elevated P2 amplitude on hits compared to FA trials, supports that this amplification cannot solely be accounted for in terms of licking-related activity. Thus, we conclude that neural activity underlying the P2 ERP peak is involved in detection of the target tone. We also observed a non-significant trend toward larger P2 amplitude on hit trials than misses, which is consistent with the target detection interpretation of the P2 function. We did not observe any significant difference between the frontal and parietal P2 amplitudes.

In principle the P2 might have been larger in amplitude on hit trials than CRs or FAs due to the target pitch (3000 Hz) intrinsically (i.e., independent of target detection or attentional state) eliciting a larger P2 response than the distractor pitch (1500 Hz), despite their identical physical intensity. However, as there was no significant difference between the P2 on miss trials (when the target pitch was presented) and either CRs or FAs (when the distractor was presented), significant pitch “tuning” in the P2 response is unlikely. Furthermore, recordings in two control animals during the equally probable passive presentation of the two pitches revealed a significant trend toward a larger P2 amplitude in response to the low pitch as compared to the higher pitch. While these data are not sufficient to establish frequency tuning in the rat P2 component, tuning following the trend in our data would reduce the relative P2 amplification on hit trials that we observed. Consistent with these considerations, an investigation of frequency tuning of the auditory ERP in rats did not report significant tuning of this auditory ERP component (Knight et al., 1985). We thus conclude the P2 amplification we observed on hit trials most likely reflects target detection related neural activity.

This conclusion is comparable to findings from surface recordings in rats (Shinba, 1997; Ahnaou et al., 2018) which show a P2 with a parietal maximum that was larger in response to target than distractor tones in a head-fixed auditory active oddball task. This refers to a go/no-go task in which the targets are relatively rare. Similar observations have been reported by others in anterior cingulate cortex and hippocampus, but without statistically comparing P2 amplitude on different trial types (Hattori et al., 2010). However, in another study, skull recordings at the vertex failed to show a significant difference between P2 amplitudes in response to targets and standards in an auditory active oddball task (Sambeth et al., 2003).

Regardless, as target trials were rare in these previous studies, oddball amplification of responses to rare tones was conflated with effects of target detection processes during the active task. Indeed, frontal and parietal cortical rare-tone amplification effects have been reported in rats at multiple ERP peaks including the P2 (Imada et al., 2013). In contrast to these previous studies, the target and distractor stimuli were presented with equal probability in our auditory go/no-go task, so a rare-tone amplification effect is ruled out. Thus, to our knowledge the present results are the first to specifically implicate the rat P2 in target detection as distinct from response activation processes during an auditory go/no-go task.

The P2 enhancement we observed during hit trials in rat frontal and parietal cortex appears analogous to the go-P2 around 200 ms post-stimulus reported in recent equiprobable auditory go/no-go ERP experiments in humans (Borchard et al., 2015) and other auditory go/no-go studies (Gajewski and Falkenstein, 2013). The human frontal-parietal P2 peak was larger during go trials and could thus be associated with response activation or target detection as we found in the rat.

### The N2 negativity does not index response inhibition but may reflect error processing

In our dataset the N2 amplitude was not consistently associated with active response inhibition on no-go trials. We instead found that the N2 amplitude is larger on miss than CR trials, which might indicate error-related cortical processing. This interpretation is dubious in our dataset because our rats were relatively proficient at the task, resulting in fewer incorrect than correct trials in each session, leading to a significantly greater variance in our incorrect trial ERPs than correct trial ERPs. Greater variability could be reflected as a bias toward systematically larger peaks in the miss trial ERPs. Thus, while our results are consistent with the possibility that the N2 negativity reflects processing or monitoring of behavioral errors, we cannot rule out a contribution from bias due to different-sized samples of each trial type.

The N2 amplitude has been associated with error-processing in some human ERP experiments. In this context the component is known as the error-related N2 or error negativity (Ne; Falkenstein et al., 1991; Scheffers et al., 1996; Botvinick et al., 1999; Kok et al., 2004). The N2 response we observed in rats may be analogous to these error-related negativities described in human ERPs. Future experiments may be able to address this issue more decisively if more difficult tasks are used to elicit greater numbers of error trials.

In humans, multiple go/no-go ERP studies using visual or auditory stimuli have reported a frontal-central no-go N2 around 200–400 ms post-stimulus that is enhanced during no-go relative to go trials (Pfefferbaum et al., 1985; Jodo and Kayama, 1992; Sasaki et al., 1993; Watanabe et al., 2002; Bekker et al., 2005a,b; Kaiser et al., 2006; Smith et al., 2007; Borchard et al., 2015). Because the no-go N2 was present even when targets on go trials were merely counted mentally rather than their presence being reported with an overt behavioral response (Pfefferbaum et al., 1985; Smith et al., 2008), it may reflect “cognitive control” (Folstein and Van Petten, 2008) or “cognitive inhibition” in auditory (Smith et al., 2008) and visual go/no-go tasks (Gajewski and Falkenstein, 2013), rather than motor inhibition. Again, our results do not support that such a process occurs in the rat brain, at least in the context of equiprobable target and distractor stimuli.

### The (earlier) P3E peak does not reflect target detection, consistent with a previously identified role in deviance detection

Previous rat work reporting a P3 response around 200%, 250 ms (Yamaguchi et al., 1993; Imada et al., 2013), suggest this component reflects novelty or deviance analogously to the human P3a (Linden, 2005; Polich, 2007). However, other rat studies have reported a go-related P3 with peak latencies overlapping the novelty-related P3 but ranging to later times up to or exceeding 400 ms post-stimulus (Shinba, 1997; Sambeth et al., 2003; Hattori et al., 2010). We therefore defined separate early and late P3 peaks.

Neither stimulus in our experiments was rare compared to the other, so we expect no novelty, deviance, or mismatch-related contribution to the P3E in our experiments. This task design allowed us to attribute a role in target detection to the P2. In contrast to the P2, we did not find support for the P3E having a role in target detection. Rather, we noted that the P3E amplitude on miss trials was significantly larger than on CR trials. While this might reflect error processing after failed go trials, this interpretation is subject to the same concern as the previously discussed N2 error-related results, as the miss ERPs are based on fewer trials than the CR ERP from the same session.

### The (later) P3L peak does not index target detection, response inhibition or production

We observed a significantly larger P3L on misses than FAs, arguing against a role of the P3L in response production. The P3L was also significantly larger on misses than CRs, and not significantly different on hits and misses, arguing against a role of the P3L in target detection. As previously mentioned, interpreting the larger P3L amplitude on miss trials as an indication of error-processing is subject to the concern that error trials were fewer and their associated ERPs more variable, likely resulting in a bias toward larger peak amplitudes on incorrect trials.

Previous auditory ERP studies in the rat have generally presented ERPs showing a larger target P3 than distractor P3, which in some cases may not be present at all (Shinba, 1997; Sambeth et al., 2003; Hattori et al., 2010; Ahnaou et al., 2018). These studies used oddball paradigms meaning that target tones were rare compared to distractors. Their results are consistent with the P3 playing a role in target detection or response production, but since the target tones were rare compared to distractors in these studies, interpretation of the P3 is confounded by potential oddball responses to the rare target tones.

Similarly, multiple human studies (Pfefferbaum et al., 1985; Rockstroh et al., 1996; Ochoa and Polich, 2000) describe a P3 peak around 300 ms post-stimulus with a parietal or parietal-central maximum that is larger during target than standard trials in oddball tasks. In equiprobable go/no-go tasks this P3 has been associated with response production (Pfefferbaum et al., 1985; Borchard et al., 2015), as well as target detection in auditory mental counting experiments without direct motor responses (Picton and Hillyard, 1974; Wronka et al., 2008).

Our results do not support a role for either the early or late P3 peak in target detection. Rather they suggest that the larger P3 on target trials noted in previous rat work could be due to an oddball amplification of the target response as opposed to target detection. Our P3 results are consistent with a study of parietal ERPs in rats performing a visual sustained attention task, which found a “P300” response (peaking before ∼250 ms post-target onset) that was sensitive to target stimulus duration but not to detection (i.e., no hit-miss difference) or attentional load (Broussard and Givens, 2010). They concluded that the parietal P3 reflected “sensory aspects of the target, and not detection per se.”

### General comparison to human ERPs

In terms of comparing the phenomenology of rat auditory ERPs to humans’, Sambeth et al. (2003) considered the rat P1, N1, P2, N2, and P3 peaks as corresponding to human ERP components simply according to their order of occurrence and polarity, and found the peak latencies in the rat to be 1.8 times earlier in the rat as compared to humans in roughly matched rat and human auditory active oddball paradigms—except for the P3 which occurred at roughly the same latency in rats and humans (350–380 ms in that study). On this basis they suggested the earlier peaks reflect “sensory processes” while the P3 reflects a more “cognitive…elaborative processing of stimuli” that is amplified by the expectation of reward in the active task. This is consistent with the demonstration of rare-tone response enhancements of the P3E in rats that could not be accounted for by simple stimulus-specific adaptation mechanisms (Imada et al., 2013), suggesting some more sophisticated memory comparison at the P3E between rare oddball tones and the recent history of standard tones.

As discussed previously, the human ERP literature suggests the involvement of the P2 and P3 ERP components in target detection and response production, and potential involvement of the N2 and P3 components in response inhibition. While our recordings from rat brain support the role of the P2 peak specifically in target detection, our results do not support a major role of the P3 in target detection. Rather, our results are more consistent with those of Broussard and Givens (2010) who reported a parietal P3 response in a visual task that was not modulated by attentional load and was virtually the same on hit and miss trials. Thus, despite the identification of P3 responses in rat brain that share some properties with their human analogues, the rat P3 responses do not appear to share the role in target detection or response production that has been reported for the human P3.

Similarly, our results do not reveal any obvious analog in the rat of no-go N2 or no-go P3 responses described in some human studies. We cannot decisively conclude at this stage whether these differences represent real species differences or differences in task context. These no-go peaks might emerge in rats, as in humans (Nieuwenhuis et al., 2004), when targets are relatively frequent and distractors are rare, leading to greater priming of the go response and more difficulty or conflict in attempting to inhibit the go response.

### Induced oscillatory contributions to ERPs

The similar latency of P3 responses in humans and rats in contrast to the earlier evoked potential components recalls the suggestion that oscillatory brain mechanisms may be tuned to maintain a similar set of frequencies across species from rodents to humans (Buzsáki et al., 2013), and raises interesting questions about how stimulus-induced oscillations may be related to ERP components (Broussard and Givens, 2010; Ahnaou et al., 2018). Our spectral analysis found that post-stimulus induced low-frequency power (1–7 Hz) was greater on hit trials than CRs, especially in frontal cortex, whereas power between 8 and 14 Hz was suppressed on hit relative to CRs. We also calculated average coherence between frontal and parietal electrodes as a function of time during each trial, and observed broadband coherence induced on hit trials as compared to CRs or misses. The induced low-frequency power and broadband interarea coherence on hit trials could reflect detection-related activity but are likely to also reflect rhythmic licking for water.

Comparing miss trials to CRs and hits with our spectral analysis revealed a band near 7 Hz at which power was particularly elevated on miss trials, even before the onset of the tone. Since we observed greater N2, P3E, and P3L amplitudes on miss trials compared to CRs, it is possible that our P3 and N2 ERP results reflect error-related oscillatory activity in the θ range as has been reported in the context of a delayed reaction-time task (Narayanan et al., 2013; Laubach et al., 2015), particularly since our P3L definition allowed later peaks (up to 1000-ms latency) than most previous studies (typically not later than 500-ms latency). However, our observation of elevated θ-power before the unpredictably timed tone on miss trials suggests instead a spontaneous state of reduced attention that predisposes the animal toward failing to respond to, i.e. missing, the target tone. This is consistent with results from a study of cortical oscillatory dynamics in rats performing a simple auditory detection task with unpredictably timed target tones (Herzog et al., 2014). However, that latter study found that both increased low-frequency power and interarea (frontal-parietal) coherence predicted miss trials, whereas our pre-stimulus result was specific to the local synchronization (i.e., power) and did not appear in the interarea coheregrams. This difference between studies may reflect a heightened state of sustained attention during our more complicated discrimination task, resulting in less global low-frequency synchronization (i.e., interarea coherence) even during the animal’s least attentive moments during a session, just before miss trials.

## Conclusions

Frontal and parietal ERPs corroborate the existence of distinct P3E and P3L peaks in rats, as has been previously reported. We found detection of the target tone to be indexed by a larger P2 peak in frontal and parietal cortex on hit trials. The P2 amplitude does not solely reflect preparation of a motor response. This is the main result of the present study and appears to support consistent functions of the P2 in rats and humans. We did not observe any clear correlate of target detection or response inhibition at the N1, N2, P3E, or P3L peaks. This is in contrast to human studies suggesting P3 involvement in target detection and motor response inhibition, and N2 involvement in cognitive response inhibition. Trends towards larger N2, P3E, and P3L amplitudes on miss trials than CRs may indicate neural processing of behavioral errors.

These results complement previous work in rats using active oddball tasks, as our experiments did not confound potential rare-tone response amplifications (“oddball responses”) with other factors that modulate peak amplitudes. Our quantification of task-related modulation of rat equiprobable go/no-go ERPs constrains potential cortical mechanisms of auditory discrimination. Moreover, our study of invasive multi-site FP recordings during active auditory discrimination behavior contributes to the growing literature showing that the rat brain can be used to probe neural processes of sensation, perception, and cognitive control in ways that are often more feasible and less expensive in rats than in humans or non-human primates.

## Synthesis

Reviewing Editor: Tatyana Sharpee, The Salk Institute for Biological Studies Decisions are customarily a result of the Reviewing Editor and the peer reviewers coming together and discussing their recommendations until a consensus is reached. When revisions are invited, a fact-based synthesis statement explaining their decision and outlining what is needed to prepare a revision will be listed below. The following reviewer(s) agreed to reveal their identity: Aaron Gruber.

This manuscript investigates potential analogues between human ERP correlates in a go/no-go tasks and rat field potential correlates in a similar task. The researchers recorded from the medial-dorsal frontal and parietal cortex as rats completed an auditory discrimination task. They report that the P2 response was analogous to that in human ERP. The authors also found the interesting differences between the N2 and P3 in the rats in comparison to humans. The manuscript advances the field, because there are few other papers demonstrating animal analogues of human EEG responses.

There are three main concerns that need to be addressed before publication. First, the location of the probes is not clear and no histology has been done. Second, questions were raised about the experimental set up and additional analyses that would be necessary. Finally, the reviewers noted a poor writing level throughout the manuscript. Editing from an established professional or a senior mentor is advised to prepare the manuscript for re-submission. Several suggestions are provided below. A lot of enthusiasm for the findings was lost over the course of reading the manuscript. Some sentences are rather long and interspersed with numerous citations, requiring a significant working memory to understand in its entirety.

Missing methodological details:

The paper does not show any histology. Because field potentials largely reflect synaptic inputs to these structures, and the source of inputs is heterogeneous across layers, it is important to know where the recording sites are. The paper could also benefit from a correlation between the signaling results and recording depth.

Related to the above point, the anatomical location of the probes is unclear. The authors implanted large arrays but report only a single frontal and single parietal response. A related point is that there is an outlier animal with a representative response very different than the others. In my opinion if the other nine animals have similar responses, then the authors would be best served to re-analyze the data and rewrite the manuscript without the outlier data. This may be a function of bad placement in one animal.

The paper refers to the signals as local field potentials, but does not specify the referencing for recording. Are they referenced to skull or to other electrodes in the same structure? If skull, it is more appropriately described as a field potential without the specification of local. If the authors insist on this terminology, they should at least acknowledge the inaccuracy up front in the paper.

The manuscript indicated that some animals could not learn the task. What was the attrition rate? How many sessions were required for task acquisition?

What was the diameter of the electrode wire, how much of the tip was exposed, what was typical impedance? This is important in order to assess signal amplitudes.

In the table, the rightward column is labeled as 'power', but text indicates that it is a p value. It should instead report power or Beta.

The description of artifact removal was not very detailed, and seems liberal. Were there any artifacts around the analysis window. Presumably the cues are evoking movement, and so some electrical artifacts may be present.

Comments on analysis:

It would be helpful to justify the equiprobable Go-NoGo task. It is not enough to discuss future directions or possibilities of treatment, what is the issue at hand, that is the generators of these attentional electrophysiological response in the study and what do they mean for cognition? Without a clear plan in the outset the interpretation can be difficult.

The grand averages in Fig 2 & 3 appear to have a surprising amount of high-frequency signal, considering there were 2 levels of averaging. Please show typical examples of single-trial traces. Ideally show several superimposed so we can see the variance. The amplitude of the ERP events appear small, so one worries about leak of events (or artifacts) from the high-pass part of the filter.

Are the ERP related to the onset of events within particular frequency bands? At least minimal analysis in the time-frequency domain should be added to address this. The authors could also investigate phase coherence across electrodes or across trials. This would help get to the bottom of what is driving the signal apparent in the average.

Introduction: The statements within the first paragraph, while true, are not germane to the current study as you do not demonstrate response inhibition neural correlates. Please rewrite with relevant neurophysiological correlates of attention specific to your study (i.e. target detection). The final sentence of the introduction indicates that you wanted to test for response inhibition using the equiprobable model. However, you interpret the lack of a response inhibition correlate as due to the equiprobability of the design. Suggesting that rare-tones would amplify a response inhibition correlate. If that were the case why did you not use a standard oddball design to search for oddball responses?

Methods: The methods section is lacking detail. It appears that 32 electrode arrays are implanted in both the frontal and parietal cortex. However it was not clear how many channels are being used to record and what is the success rate. Are 3 channels from the frontal and 10 from the parietal? It would be important to convey this and specify whether channels are being averaged within each animal to generate an ERP. Given this many electrodes, one would expect a comparison of anterior-posterior or medial to lateral expression of each array, rather than merely a frontal and parietal representation.

Results: The results were disorganized and need a thorough rewrite from top to bottom. The first two pages of the results are spent explaining the different components of the ERP response. This is better to put in the introduction section and focus on the results. There are many problems with the language. (e.g. line 181, “Following the EEG literature” [one would hope so], line 196 “We call P3E”) Well of course there is a literature of ERP data but you can open up a discussion of that literature by explaining how some consensus over ERP components have been demonstrated over the decades. Condense these paragraphs and refer to the establishment of these correlates specifically in the papers that are relevant to target detection.

Another example line 194 “a mid-latency negativity we called N2”. Have you consulted the literature on the N2 response and what behaviors it may correlate with?

Attempt to make the results section a story that an interested scientist would look forward to reading rather than a rote recitation of facts.

For the results please go in the order that the ERP events occur. The main finding is the P2 but we can highlight this with a header.

The paragraph on page 19 is spent discussing data that were not significantly different in a way that it would be if it were.

At no point do you mention how many Miss trials and FA trials there are. As such, it is hard to interpret these data. If we know that there are fewer than 10 miss trials we can assume that it will be difficult to make statistical comparisons to CR and Hit trials. Much time is spent in this discussion trying to interpret the miss data, but this emphasis is misplaced if there are few trials.

Discussion: Once the other revisions are done a proper discussion can be written. In the summary and conclusion page the “disappointment” should be deleted. You mention at no point in the discussion the neural processing of reward prediction errors, and so it should not be in the summary.

It is not clear why the authors did not further discuss the error-related negativity analogy of their N2 results. The authors discuss N2 amplitude as associated with error-processing in human ERP, and show their N2 amplitude recorded in rats was larger on miss trials. Yet, on line 384, they state future experiments may be able to address the analogy more decisively via more difficult tasks. The error-related negativity is a major component of the human ERP in decision-making. This lack of confidence in analogy between the ERN and the N2 described by the authors here warrants further discussion.

Minor edits:

There are many grammatical errors that make reading difficult.

The authors use the unnecessary adverb “broadly” throughout the manuscript (such as: “broadly support” and “broadly consistent”). The paper would benefit from removal of these words.

Line 12 change “revealed” to “indicated”

Line 13 change “is related” to “precedes”

Line 43 change stimulation contexts to target or non-target tone.

Line 55 change later to “more recent”

Line 65 re-write this sentence as I have no idea what an “automatic rare-tone response amplification” is. Related to oddball response?

Line 199 and throughout, if you have two parenthetical statements, combine them into 1, i.e. (~400 ms; Sambeth etc.)

Line 216 Omit “Neverthelesss”

Line 224 Move the ANOVA sentence up near the beginning of the Hit vs. CR peak amplitudes header.

Include early in this paragraph a clause like: “The main effect of Trial Type indicated that detection of signals amplified the P2 signals compared to CR. ”

P244 change “beep” to “tone” please find and replace.

Line 278 “tended weakly” “those these differences are not statistically significant” there is no difference period. Do not mislead the reader.

Discussion

Line 305 “We observed an amplification of the P2 amplitude on hit trials as compared to correct rejections*CRS)” Instead use common approach, “We observed that detection of the target tone increase the P2 amplitude...”

Similarly line 309 “the P2 amplification on hit trials” change to “detection-related P2 responses”

Line 458-459 “On this view the earlier peaks are earlier because the rat brain is smaller” Please omit this sentence unless you plan to mathematically back it up. For example the conductance velocity of an auditory axon is between 10-20 m/s and calculate the amount of msec it takes to travel the distance. Likely this is not a proper explanation.

Line 465 discusses the lack of a P3 effect, better to focus more on the P2 effect which is positive.

Line 476 this data makes me question whether the oddball paradigm may be better for finding response inhibition correlates.

The sentence on lines 482 - 484 is not clear.

Comments on figures:

The use of box plots is excellent - more papers should use these.

Figure 1. Can you show a percent correct summary of the entire group?

Figure 2 related to above. Is there only one outlier? If so what does the data look like if omitted?

Figure 3 the summary panels on the right look very small and what do the plus signs mean here? In figure 3 and further indicate significant differences somehow (asterisk) and in the legend include at least basic statistics and point to this with explicit statements.

The fonts on plot axes are inconsistent, and some are miniscule (Figure 5).

The varying y-axes within each figure are confusing - these should each use the same scale where possible (e.g. Figure 2).

Comments on the visual abstract:

The Abstract summary figure can be significantly improved. Please indicate the delay between the tone and response time if any and mark the responses. Are they at 500 ms? The right have does not appear to convey interesting information. It might be better to focus on the results on the left half in more detail. Which part of the brain is the P2 recorded from? Also, considering taking the auditory cue out of the white bounding box. Details of cue type can be displayed.

## References

[B1] Ahnaou A, Biermans R, Drinkenburg WHIM (2018) Cholinergic mechanisms of target oddball stimuli detection: the late “P300-like” event-related potential in rats. Neural Plast 2018:4270263. 10.1155/2018/4270263 30410536 PMC6206555

[B2] Bekker EM, Kenemans JL, Hoeksma MR, Talsma D, Verbaten MN (2005a) The pure electrophysiology of stopping. Int J Psychophysiol 55:191–198. 10.1016/j.ijpsycho.2004.07.005 15649550

[B3] Bekker EM, Kenemans JL, Verbaten MN (2005b) Source analysis of the N2 in a cued Go/NoGo task. Brain Res Cogn Brain Res 22:221–231. 10.1016/j.cogbrainres.2004.08.011 15653295

[B4] Bledowski C, Prvulovic D, Hoechstetter K, Scherg M, Wibral M, Goebel R, Linden DE (2004) Localizing P300 generators in visual target and distractor processing: a combined event-related potential and functional magnetic resonance imaging study. J Neurosci 24:9353–9360. 10.1523/JNEUROSCI.1897-04.2004 15496671 PMC6730097

[B5] Bokil H, Purpura K, Schoffelen J-M, Thomson D, Mitra P (2007) Comparing spectra and coherences for groups of unequal size. J Neurosci Methods 159:337–345. 10.1016/j.jneumeth.2006.07.011 16945422

[B6] Borchard JP, Barry RJ, De Blasio FM (2015) Sequential processing in an auditory equiprobable Go/NoGo task with variable interstimulus interval. Int J Psychophysiol 97:145–152. 10.1016/j.ijpsycho.2015.05.010 26024616

[B7] Botvinick M, Nystrom LE, Fissell K, Carter CS, Cohen JD (1999) Conflict monitoring versus selection-for-action in anterior cingulate cortex. Nature 402:179–181. 10.1038/46035 10647008

[B8] Broussard JI, Givens B (2010) Low frequency oscillations in rat posterior parietal cortex are differentially activated by cues and distractors. Neurobiol Learn Mem 94:191–198. 10.1016/j.nlm.2010.05.006 20493272

[B9] Buzsáki G, Logothetis N, Singer W (2013) Scaling brain size, keeping timing: evolutionary preservation of brain rhythms. Neuron 80:751–764. 10.1016/j.neuron.2013.10.002 24183025 PMC4009705

[B10] Courchesne E, Hillyard SA, Galambos R (1975) Stimulus novelty, task relevance and the visual evoked potential in man. Electroencephalogr Clin Neurophysiol 39:131–143. 10.1016/0013-4694(75)90003-6 50210

[B11] Crowley KE, Colrain IM (2004) A review of the evidence for P2 being an independent component process: age, sleep and modality. Clin Neurophysiol 115:732–744. 10.1016/j.clinph.2003.11.021 15003751

[B12] Falkenstein M, Hohnsbein J, Hoormann J, Blanke L (1991) Effects of crossmodal divided attention on late ERP components. II. Error processing in choice reaction tasks. Electroencephalogr Clin Neurophysiol 78:447–455. 10.1016/0013-4694(91)90062-9 1712280

[B14] Folstein JR, Van Petten C (2008) Influence of cognitive control and mismatch on the N2 component of the ERP: a review. Psychophysiology 45:152–170. 10.1111/j.1469-8986.2007.00602.x 17850238 PMC2365910

[B15] Gajewski PD, Falkenstein M (2013) Effects of task complexity on ERP components in Go/Nogo tasks. Int J Psychophysiol 87:273–278. 10.1016/j.ijpsycho.2012.08.007 22906814

[B16] Hattori M, Onoda K, Sakata S (2010) Identification of rat P3-like processes in the anterior cingulate cortex and hippocampus. Neurosci Lett 472:43–46. 10.1016/j.neulet.2010.01.052 20117173

[B17] Herzog L, Salehi K, Bohon KS, Wiest MC (2014) Prestimulus frontal-parietal coherence predicts auditory detection performance in rats. J Neurophysiol 111:1986–2000. 10.1152/jn.00781.2012 24572093 PMC4044341

[B18] Imada A, Morris A, Wiest M (2013) Deviance detection by a P3-like response in rat posterior parietal cortex. Front Integr Neurosci 6:127. 23316147 10.3389/fnint.2012.00127 PMC3539781

[B19] Jodo E, Kayama Y (1992) Relation of a negative ERP component to response inhibition in a Go/No-go task. Electroencephalogr Clin Neurophysiol 82:477–482. 10.1016/0013-4694(92)90054-l 1375556

[B20] Kaiser S, Weiss O, Hill H, Markela-Lerenc J, Kiefer M, Weisbrod M (2006) N2 event-related potential correlates of response inhibition in an auditory Go/Nogo task. Int J Psychophysiol 61:279–282. 10.1016/j.ijpsycho.2005.09.006 16298004

[B21] Knight RT, Brailowsky S, Scabini D, Simpson GV (1985) Surface auditory evoked potentials in the unrestrained rat: component definition. Electroencephalogr Clin Neurophysiol 61:430–439. 10.1016/0013-4694(85)91035-1 2412796

[B22] Kok A, Ramautar JR, De Ruiter MB, Band GP, Ridderinkhof KR (2004) ERP components associated with successful and unsuccessful stopping in a stop-signal task. Psychophysiology 41:9–20. 10.1046/j.1469-8986.2003.00127.x 14692996

[B23] Laubach M, Caetano MS, Narayanan NS (2015) Mistakes were made: neural mechanisms for the adaptive control of action initiation by the medial prefrontal cortex. J Physiol Paris 109:104–117. 10.1016/j.jphysparis.2014.12.001 25636373 PMC5292776

[B24] Linden DE (2005) The p300: where in the brain is it produced and what does it tell us? Neuroscientist 11:563–576. 10.1177/1073858405280524 16282597

[B25] Narayanan NS, Cavanagh JF, Frank MJ, Laubach M (2013) Common medial frontal mechanisms of adaptive control in humans and rodents. Nat Neurosci 16:1888–1895. 10.1038/nn.3549 24141310 PMC3840072

[B26] Nieuwenhuis S, Yeung N, Cohen JD (2004) Stimulus modality, perceptual overlap, and the go/no-go N2. Psychophysiology 41:157–160. 10.1046/j.1469-8986.2003.00128.x 14693011

[B27] Ochoa CJ, Polich J (2000) P300 and blink instructions. Clin Neurophysiol 111:93–98. 10.1016/s1388-2457(99)00209-6 10656515

[B28] Paxinos G, Watson C (1997) The rat brain in stereotaxic coordinates. San Diego: Academic Press.

[B29] Pek J, Park J (2019) Complexities in power analysis: quantifying uncertainties with a Bayesian-classical hybrid approach Psychol Methods 24:590–605. 30816728 10.1037/met0000208

[B31] Pfefferbaum A, Ford JM, Weller BJ, Kopell BS (1985) ERPs to response production and inhibition. Electroencephalogr Clin Neurophysiol 60:423–434. 10.1016/0013-4694(85)91017-X 2580694

[B32] Picton TW, Hillyard SA (1974) Human auditory evoked potentials. II. Effects of attention. Electroencephalogr Clin Neurophysiol 36:191–199. 10.1016/0013-4694(74)90156-4 4129631

[B33] Polich J (2007) Updating P300: an integrative theory of P3a and P3b. Clin Neurophysiol 118:2128–2148. 10.1016/j.clinph.2007.04.019 17573239 PMC2715154

[B34] Rockstroh B, Müller M, Heinz A, Wagner M, Berg P, Elbert T (1996) Modulation of auditory responses during oddball tasks. Biol Psychol 43:41–55. 10.1016/0301-0511(95)05175-9 8739613

[B35] Sambeth A, Maes JH, Van Luijtelaar G, Molenkamp IB, Jongsma ML, Van Rijn CM (2003) Auditory event-related potentials in humans and rats: effects of task manipulation. Psychophysiology 40:60–68. 10.1111/1469-8986.00007 12751804

[B36] Sasaki K, Gemba H, Nambu A, Matsuzaki R (1993) No-go activity in the frontal association cortex of human subjects. Neurosci Res 18:249–252. 10.1016/0168-0102(93)90062-U 8127474

[B37] Scheffers MK, Coles MG, Bernstein P, Gehring WJ, Donchin E (1996) Event-related brain potentials and error-related processing: an analysis of incorrect responses to go and no-go stimuli. Psychophysiology 33:42–53. 10.1111/j.1469-8986.1996.tb02107.x 8570794

[B38] Shinba T (1997) Event-related potentials of the rat during active and passive auditory oddball paradigms. Electroencephalogr Clin Neurophysiol 104:447–452. 10.1016/S0168-5597(97)00047-6 9344081

[B39] Shinba T (1999) Neuronal firing activity in the dorsal hippocampus during the auditory discrimination oddball task in awake rats: relation to event-related potential generation. Brain Res Cogn Brain Res 8:241–250. 10.1016/S0926-6410(99)00026-9 10556602

[B41] Smith JL, Johnstone SJ, Barry RJ (2007) Response priming in the Go/NoGo task: the N2 reflects neither inhibition nor conflict. Clin Neurophysiol 118:343–355. 10.1016/j.clinph.2006.09.027 17140848

[B42] Smith JL, Johnstone SJ, Barry RJ (2008) Movement-related potentials in the Go/NoGo task: the P3 reflects both cognitive and motor inhibition. Clin Neurophysiol 119:704–714. 10.1016/j.clinph.2007.11.042 18164657

[B43] Squires KC, Squires NK, Hillyard SA (1975a) Decision-related cortical potentials during an auditory signal detection task with cued observation intervals. J Exp Psychol Hum Percept Perform 1:268–279. 10.1037//0096-1523.1.3.268 1202150

[B44] Squires KC, Squires NK, Hillyard SA (1975b) Vertex evoked potentials in a rating-scale detection task: relation to signal probability. Behav Biol 13:21–34. 10.1016/S0091-6773(75)90748-8 1111506

[B46] Watanabe N, Hirai N, Maehara T, Kawai K, Shimizu H, Miwakeichi F, Uchida S (2002) The relationship between the visually evoked P300 event-related potential and gamma band oscillation in the human medial and basal temporal lobes: an electrocorticographic study. Neurosci Res 44:421–427. 10.1016/S0168-0102(02)00159-1 12445629

[B47] Wronka E, Kaiser J, Coenen AM (2008) The auditory P3 from passive and active three-stimulus oddball paradigm. Acta Neurobiol Exp (Wars) 68:362–372. 18668159 10.55782/ane-2008-1702

[B48] Yamaguchi S, Globus H, Knight RT (1993) P3-like potential in rats. Electroencephalogr Clin Neurophysiol 88:151–154. 10.1016/0168-5597(93)90066-x 7681756

